# Interaction and Metabolic Pathways: Elucidating the Role of Gut Microbiota in Gestational Diabetes Mellitus Pathogenesis

**DOI:** 10.3390/metabo14010043

**Published:** 2024-01-10

**Authors:** Lindong Mao, Biling Gao, Hao Chang, Heqing Shen

**Affiliations:** 1 State Key Laboratory of Infectious Disease Vaccine Development, Xiang An Biomedicine Laboratory & State Key Laboratory of Molecular Vaccinology and Molecular Diagnostics, School of Public Health, Xiamen University, Xiamen 361102, China; maolindong@stu.xmu.edu.cn (L.M.); gaobiling@stu.xmu.edu.cn (B.G.); changhaomph@stu.xmu.edu.cn (H.C.); 2 Department of Obstetrics, Women and Children’s Hospital, School of Medicine, Xiamen University, Xiamen 361003, China

**Keywords:** intestinal microbiota, gestational diabetes mellitus, metabolic disorders, metabolomics

## Abstract

Gestational diabetes mellitus (GDM) is a complex metabolic condition during pregnancy with an intricate link to gut microbiota alterations. Throughout gestation, notable shifts in the gut microbial component occur. GDM is marked by significant dysbiosis, with a decline in beneficial taxa like *Bifidobacterium* and *Lactobacillus* and a surge in opportunistic taxa such as *Enterococcus*. These changes, detectable in the first trimester, hint as the potential early markers for GDM risk. Alongside these taxa shifts, microbial metabolic outputs, especially short-chain fatty acids and bile acids, are perturbed in GDM. These metabolites play pivotal roles in host glucose regulation, insulin responsiveness, and inflammation modulation, which are the key pathways disrupted in GDM. Moreover, maternal GDM status influences neonatal gut microbiota, indicating potential intergenerational health implications. With the advance of multi-omics approaches, a deeper understanding of the nuanced microbiota–host interactions via metabolites in GDM is emerging. The reviewed knowledge offers avenues for targeted microbiota-based interventions, holding promise for innovative strategies in GDM diagnosis, management, and prevention.

## 1. Introduction

Gestational diabetes mellitus (GDM) refers to glucose intolerance that results in hyperglycemia during pregnancy [1]. GDM often occurs at 24–28 weeks of gestation, but can also present earlier [2]. The pathogenesis of GDM is complex, and may be associated with obesity, insulin resistance, β-cell dysfunction, etc. [3]. The global prevalence of GDM is 13.6% and is kept increasing [4]. GDM increases the risks of maternal and neonatal complications [5]. Meta-analyses have shown that approximately 35% of women with GDM will develop type 2 diabetes mellitus (T2DM) after delivery [6].

Increasing evidence has demonstrated that gut microbiota is closely related to host metabolic health and may influence the development of various metabolic diseases, such as obesity, metabolic syndrome and T2DM. Gut microbiota dysbiosis has been considered as an important environmental factor leading to host metabolic abnormalities [7]. Gut microbes and their metabolites can affect host health by regulating host energy metabolism [8], lipid metabolism [9], bile acid homeostasis [10] and inflammatory responses [11]. Particularly during pregnancy, gut microbial imbalance has also been associated with GDM. For example, reductions in probiotics and some metabolic activity alterations of gut microbiota have been observed in GDM patients [12]. These findings suggest that modulating and optimizing gut microbial composition may represent a novel intervention strategy for GDM. However, the underlying mechanisms still need to be elucidated.

Recently, various omics technologies have applied to improve the in-depth understanding of relationship between gut microbiota and host metabolism. Microbiome techniques such as 16S rRNA gene sequencing have been widely used to portray the composition and structural features of gut microbiota [13]. Meanwhile, metabolomics as a technology of detecting all small molecule metabolites in biological samples, has been used to detect the metabolic features of the hosts’ gut microbes [14]. In particular, microbial metabolites such as short-chain fatty acids (SCFAs) and bile acids (BAs) have been evidenced to influence host physiological status by blood circulating or neural pathways, thus regulating the host health [15]. Recent studies employing quantitative metabolomics analyzed gut microbial metabolites and revealed the metabolites’ activity and function correlated with the host, which greatly advanced our knowledge of host–microbiota interactions [16,17,18]. 

Therefore, the integration of microbiome, metabolome as well and other omics approaches will facilitate an in-depth understanding of gut microbial impacts on host health and the development of microbiota-related biomarkers. In the present review, we performed a literature search to identify relevant studies on the role of gut microbiota and microbial metabolites in GDM. The search was conducted in May 2023 across the PubMed, Scopus, Web of Science, and MEDLINE databases. For the section investigating the links between gut microbiota and GDM, search terms included “gut microbiota”, “gestational diabetes mellitus”, “metabolic disorders”, “metabolomics”, “16S rRNA sequencing”, and “metagenomics”. For the microbial metabolites section, keywords used were “gestational diabetes mellitus”, “microbiota”, “metabolite”, “SCFAs”, “trimethylamine N-oxide”, “BAs”, and “amino acid metabolites”. The upcoming section on potential microbial-based interventions will utilize keywords such as “Gestational diabetes mellitus”, “Probiotic OR Prebiotic” and “Fecal microbiota transplant” to identify relevant prevention and treatment studies. We will focus on the results from the most significant studies dealing with the role of microbiota-derived metabolites in GDM.

## 2. Gut Microbiota’s Role in Gestational Diabetes Mellitus

Studies have highlighted the distinctive changes in gut microbiota in normoglycemic pregnancy. During the healthy pregnancy, a series of physiological changes occurred in hormones, immunity, and metabolism to support the well-being of both mother and developing fetus [19] Among these changes, a notable shift in insulin sensitivity, termed insulin resistance is observed [20]. Recent research has illuminated a potential nexus between insulin resistance during pregnancy and alterations in the gut microbiota composition [21]. Koren et al. [22] demonstrated the extensive remodeling of gut microbiota throughout healthy pregnancy. In a prospective cohort of 91 Finnish women, 16S rRNA sequencing revealed the first-trimester microbiota composition was similar to the non-pregnant controls. In contrast, the third-trimester microbiota was characterized by the increased interpersonal variability, reduced richness, and increased *Proteobacteria*. The third-trimester microbiota promotes metabolic changes that may support fetal growth but resemble metabolic syndrome. In summary, pregnancy induces the dramatic changes in microbiota of resembling dysbiosis, which is an inflammatory state.

Recent systematic reviews and meta-analyses of available clinical evidence suggest that metformin (MET) exposure is associated with beneficial modulation of the gut microbiome in cohorts with metabolic disease [23]. In a comparative study evaluating the effects of metformin and insulin (INS) on health parameters in GDM, significant differences were observed [24], and the study found changes in the composition of gut microbiota in the MET group, including a decrease in *Firmicutes* and *Peptostreptococcaceae*, and an increase in *Proteobacteria* and *Enterobacteriaceae*. Furthermore, metabolic profiling of the gut microbiota in the MET group revealed a prevalence of pathways associated with propionate degradation and ubiquinol biosynthesis. Mouse experiments showed modulation of the gut microbiota (by an increase in the *Akkermansia* spp. population) may contribute to the antidiabetic effects of MET, thereby providing a new mechanism for the therapeutic effect of MET in patients with T2D [25]. These findings highlight the diverse impact of MET, which extends beyond glucose metabolism to include significant effects on the composition and metabolic functions of gut microbiota. This provides insights into the potential systemic health implications of the drug [26].

**Figure 1:**
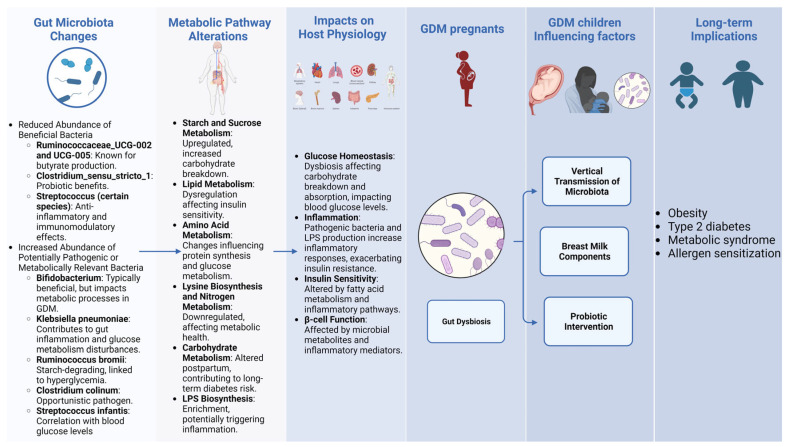
Impacts of gut microbiota and metabolic changes on gestational diabetes mellitus and long-term health outcomes.

Beneficial commensals refer to bacteria that normally reside in the human body, especially the gut, and confer health benefits to the host. Studies have shown that certain commensal bacteria like *Bifidobacterium* and *Lactobacillus* play important roles in maintaining gut barrier integrity, immune homeostasis, and metabolic health [27,28]. Specifically, the loss of beneficial commensals such as *Bifidobacterium* and *Lactobacillus* species leads to the disruption of gut barrier integrity and metabolic functions. In contrast, opportunistic pathogens are microbes that are normally harmless but can cause infectious diseases when the host immunity is compromised [29,30]. Typical opportunistic pathogens that flourish in dysbiosis comprise of the *Enterococcus* and *Staphylococcus* species. These microorganisms encourage inflammation, interrupt gut permeability, and further contribute to insulin resistance (Figure 1).

### 2.1. Early Pregnancy: Gut Microbiota, Host Overweight, and GDM Predection

In a prospective cohort of 91 Finnish women, Koren et al. [22] demonstrated the first-trimester microbiota composition was similar to the non-pregnant controls; the study had a small sample size and limited geographic/ethnic diversity, but provided initial evidence that early pregnancy microbiota may resemble normal non-pregnant microbiota. Gomez-Arango [31] et al. performed a cross-sectional study in 2016 among 70 overweight and obese pregnant women in early gestation (mean 16 weeks) in Australia. Compared to overweight women, the obese cohort exhibited increased *Actinobacteria*, decreased *Tenericutes*, and positive correlations between BMI and *Lachnospiraceae/Rikenellaceae*. The moderate sample size was limited regionally. Results indicated obesity may lead to early pregnancy dysbiosis [32]. In a study by Ma et al. [33] with fecal samples obtained from 98 GDM and 98 matched normoglycemic pregnant women at 10–15 weeks of gestation, GDM was associated with lowered alpha diversity, increased beta diversity, increased *Eisenbergiella, Tyzzerella, Lachnospiraceae NK4A136*, and decreased *Parabacteroides, Megasphaera,* and *Dialister*. The large sample size has well characterized the metabolism function of GDM versus normal pregnancy. Functional analysis revealed up-regulation of starch and sucrose metabolism pathways and down-regulation of lysine biosynthesis and nitrogen metabolism pathways in GDM. Mokkala et al. [34] conducted a prospective study in Finland in 2017 comprising 75 pregnant women. Fecal samples were collected at a mean of 12.9 weeks of gestation and gut microbiota was profiled by 16S rRNA gene V4 region sequencing. Compared to women who did not develop GDM, those with subsequent GDM exhibited a significantly elevated relative abundance of *Ruminococcaceae*. 

For summary, the early pregnancy microbial changes (alpha/beta diversity shifts, specific taxa abundance changes) associate with later GDM occurrence. In particular, increased *Ruminococcaceae* has been linked to GDM risk in multiple studies. This provides a basis for early prediction GDM by using key microbial markers. Larger, multicenter prospective studies constructing and validating microbiome-based GDM prediction models are warranted. This could enable the first trimester GDM risk assessment based on gut microbiota patterns, facilitating early prevention and intervention.

### 2.2. Second Trimester of Pregnancy: Gut Microbiota, Host GDM, and Metabolomis Pathogensis

Kuang [35] et al. used shotgun metagenomic sequencing to analyze the gut microbiota of 43 GDM patients and 81 healthy pregnant women during mid-pregnancy. GDM patients showed increased abundance of *Bifidobacterium* and *Klebsiella pneumoniae*, and decreased abundance of *Alistipes genus, Bifidobacterium genus,* and *Verrucomicrobia genus* when compared to normal glucose tolerance (NGT) women. Functional prediction analysis revealed enrichment of lipid and amino acid metabolism pathways in the GDM group. Liang et al. [36] collected fecal samples from 35 GDM patients and 25 healthy pregnant women during the second and third trimesters. 16S rRNA gene V4 region sequencing showed that compared to healthy pregnant women, GDM patients had decreased *Ruminococcaceae_UCG-002, Ruminococcaceae_UCG-005, Clostridium_sensu_stricto_1*, and *Streptococcus*, while *Bacteroides* and *Lachnoclostridium* were increased. Correlation analysis found *Paraprevotella, Roseburia, Faecalibacterium,* and *Ruminococcaceae_UCG-002* were negatively correlated with blood glucose, while *Sutterella, Oscillibacter*, and *Bifidobacterium* were positively correlated with glucagon-like peptide-1 (GLP-1) levels. Additionally, a model containing 20 gut microbial genera and glucose levels effectively distinguished between GDM and normal pregnant groups. 

Wei [37] utilized 16S rRNA gene sequencing to examine fecal samples from 15 GDM patients and 18 pregnant women with NGT. The results suggest a higher abundance of *Ruminococcus bromii*, *Clostridium colinum*, and *Streptococcus infantis* (*S. infantis*) in GDM patients in contrast to NGT. Moreover, *S. infantis* was positively correlated with glucose levels regardless of BMI adjustments. Wang [38] et al. compared the gut microbiome and metabolomic profiles between 59 pregnant women with GDM and 48 healthy pregnant controls. Correlation analysis revealed associations of specific bacterial taxa with blood glucose levels and fetal physical parameters. Furthermore, network analysis showed interactions between altered gut bacteria and perturbed metabolites involved in carbohydrate and amino acid metabolism pathways.

Generally, the microbial changes include a notable decrease in taxa typically associated with beneficial health outcomes and an increase in taxa that may be detrimental [39]. Specifically, the beneficial taxa that are reduced in GDM patients include the following: *Ruminococcaceae_UCG-002*: Known for its butyrate-producing capabilities, this bacterium is essential for maintaining gut health. *Ruminococcaceae_UCG-005*: Also a butyrate producer, its diminished levels may adversely affect gut homeostasis and integrity [40] *Clostridium_sensu_stricto_1*: Generally considered to confer probiotic benefits, the decline of this genus could play a role in the dysbiosis state associated with GDM [41]. *Streptococcus*: Some species within this genus exhibit anti-inflammatory and immunomodulatory effects, and their decreasing levels might disrupt the gut environment [42].

Conversely, potentially harmful taxa that are elevated in GDM patients include the following: *Bifidobacterium*: Typically beneficial, but in the context of GDM, it may assume a role that negatively impacts metabolic processes [35]. *Klebsiella pneumoniae*: A pathogenic bacterium, its increased abundance could contribute to gut inflammation and disturbances in glucose metabolism [43]. *Ruminococcus bromii*: A starch-degrading microbe whose heightened levels in GDM could be linked to hyperglycemia [44]. *Clostridium colinum*: While some strains are opportunistic pathogens, their precise role in GDM requires further investigation [34]. *Streptococcus infantis*: This bacterium’s positive correlation with blood glucose levels suggests a potential harmful impact in the context of GDM [45].

Taken together, the dysbiosis of the gut microbiome during mid-pregnancy may impact host energy homeostasis and glucose regulation through modulating microbial carbohydrate, lipid, and amino acid metabolism. The resulting alterations in hormone secretion, substrate availability, and nutrient signaling could undermine insulin sensitivity and pancreatic β-cell function, contributing to hyperglycemia and the pathogenesis of GDM. Further elucidation of the metabolic activities of gut microbe dysregulated in pregnancy may reveal novel diagnostic biomarkers and therapeutic targets for GDM.

### 2.3. Third Trimester of Pregnancy: Gut Microbiota Dysbiosis and Host Postpartum Diabetes Risk 

Recent studies by Ferrocino et al. [46] and Crusell et al. [47] have examined changes in the gut microbiota composition from the second/third trimester to postpartum and its associations with GDM diagnosis and future diabetes risk. Both found evidence of gut microbiota dysbiosis in the third trimester in women with GDM compared to normal glucose tolerant pregnant controls. Metagenomic prediction suggested enrichment of carbohydrate metabolism and LPS biosynthesis pathways [44]. Specifically, increases in certain gut microbes like *Faecalibacterium, Blautia, Coprococcus, Dorea,* and *Lachnospiraceae* were observed during pregnancy in women with GDM. In contrast, taxa like *Bacteroides* and *Collinsella* were decreased. Importantly, microbiota dysbiosis persisted postpartum as well in women with prior GDM [45]. For instance, Crusell et al. found 13 operational taxonomic units (OTUs) still differentially abundant between prior GDM and normal glucose regulation groups at ~8 months postpartum. This suggests an enduring impact of pregnancy-related microbial changes on the post pregnancy maternal diabetes risk.

Notably, specific microbes were linked to glucose metabolism and inflammatory markers during pregnancy. Higher *Faecalibacterium* abundance correlated with lower fasting glucose, while *Collinsella* positively associated with insulin resistance [44]. Greater *Sutterella* related to higher C-reactive protein [44]. Such relationships provide evidence that microbiota changes may directly influence GDM pathophysiology. Proposed mechanisms include microbiota-driven production of metabolites like SCFAs effects on gut barrier integrity, regulation of inflammatory signaling pathways, and modulation of insulin sensitivity [48]. Further studies are still needed to firmly establish causality between gut dysbiosis and abnormal glucose metabolism during and after pregnancy.

### 2.4. Neonatal Impact: Maternal GDM and Neonatal Microbiota

Mothers with GDM can influence the gut microbiota development of infants. Chen [49] collected fecal samples from 418 mothers (147 with GDM and 271 controls) and their newborns were collected and analyzed by 16S rRNA gene sequencing of the V3 region and metabolomics. Compared to controls, the relative abundance of *Firmicutes* increased and *Proteobacteria* decreased at the phylum level in newborns of GDM mothers. At the family level, *Streptococcaceae* was more abundant in the GDM group. Predicted metagenome functional analysis using PICRUSt2 revealed enrichment of pathways related to carbohydrate and nucleotide metabolism in the GDM group. These findings suggest maternal GDM status is associated with alterations in the neonatal gut microbiota and metabolome. Zhu [50] et al. demonstrated associations between maternal GDM, the neonatal gut microbiota, and infant BMI at 12 months of age. Analysis of meconium from 120 mother-infant pairs showed that maternal GDM was linked to reduced microbial diversity and dysbiosis in neonates. Specific bacterial genera enriched in infants of healthy mothers were negatively correlated with infant BMI. Furthermore, a co-abundant group of bacteria depleted in infants born to mothers with GDM mediated over 20% of the association between maternal GDM and increased infant BMI. These results suggest that GDM-induced dysbiosis of the newborn microbiome may contribute to increase BMI in early infancy. 

GDM mothers influence the gut microbiota of infants in two different ways, one is a vertical transmission of microbiota. GDM leads to dysbiosis in maternal microbiota, which can get transmitted to the infant gut early in life via routes like vaginal delivery and breastfeeding. GDM infants exhibit reduced microbial diversity and aberrant abundance of bacteria like *Bacteroides*, *Klebsiella*, *Staphylococcus* compared to infants of healthy mothers [51,52,53]. Wang et al. [54] found significant modifications in the oral microbiota of mothers with GDM, characterized by increased *Proteobacteria* and decreased *Firmicutes*. This altered microbiome was reflected in the neonatal gut microbiota as well, evidenced by analysis of meconium samples. Specifically, neonates born to mothers with GDM showed increased abundance of *Bacteroides, Parabacteroides, Alistipes* genera and decreased *Escherichia, Enterococcus, Lactobacillus* compared to infants of healthy mothers. Analysis of umbilical cord blood also found higher *Staphylococcus* and lower *Bifidobacterium* in neonates of GDM pregnancies [55]. The neonatal gut dysbiosis could be influenced by maternal hyperglycemia, altered nutritional status, weight gain patterns, mode of delivery and other factors related to GDM pathophysiology [51].

Breast milk plays an important role in the growth and development of infants. Breast milk can provide the nutrients that babies need, including the complex carbohydrates, proteins, antibodies, free fatty acids and hormone. Brest milk also provides a wide range of biological activities and can promote the development and maturity of the infant immune system, as well as early healthy intestinal colonization [56]. Breast milk oligosaccharides and glycoproteins, important components of the breast milk glycobiome, selectively enrich beneficial bacteria in the infant gut microbiome, with studies indicating that GDM can alter the concentration and glycosylation of these compounds in breast milk, potentially impacting the gut microbiota composition of offspring [57,58]. Ponzo et al. [53] investigated the gut microbiota composition of infants born to mothers with GDM. Fecal samples from 29 GDM infants were analyzed during the 1st week of life. Compared to their mothers, the infants showed lower microbial diversity and complexity, with dominance of *Actinobacteria* and *Proteobacteria*. The infant microbiota composition was more associated with early pregnancy maternal diet than late pregnancy diet. Notably, maternal saturated fat intake was inversely correlated with *Rikenellaceae* and *Ruminococcus* abundance in infants. Breastfed infants harbored higher *Bifidobacterium*, while formula-fed had increased *Firmicutes* diversity. Compared to healthy infants, the GDM infants exhibited dysbiosis with higher abundance of pro-inflammatory *Escherichia* and *Parabacteroides*.

Probiotic intervention in pregnant mothers GDM may foster the development of a healthy intestinal microbiota in infants [59], which further implied the maternal–infant transmission. There is research indicating that treatment of GDM significantly lowers the risk of various adverse pregnancy outcomes, including fetal macrosomia, large-for-gestational-age births, and gestational hypertension, with some evidence also pointing to a reduction in perinatal/neonatal mortality and birth trauma. Furthermore, studies on probiotics for GDM prevention show promising results, including lower GDM diagnosis rates and reduced birth weights in infants.

Overall, these studies underscore the significant influence of maternal GDM on the vertical transmission of microbiota and the development of the neonatal gut microbiome. The observed dysbiosis in infants born to mothers with GDM, influenced both by vertical transmission and early feeding practices, points to a potential pathway through which GDM may predispose infants to altered metabolic trajectories and health outcomes later in life, including obesity, type 2 diabetes, metabolic syndrome, and allergen sensitization [60,61].

**Figure 2:**
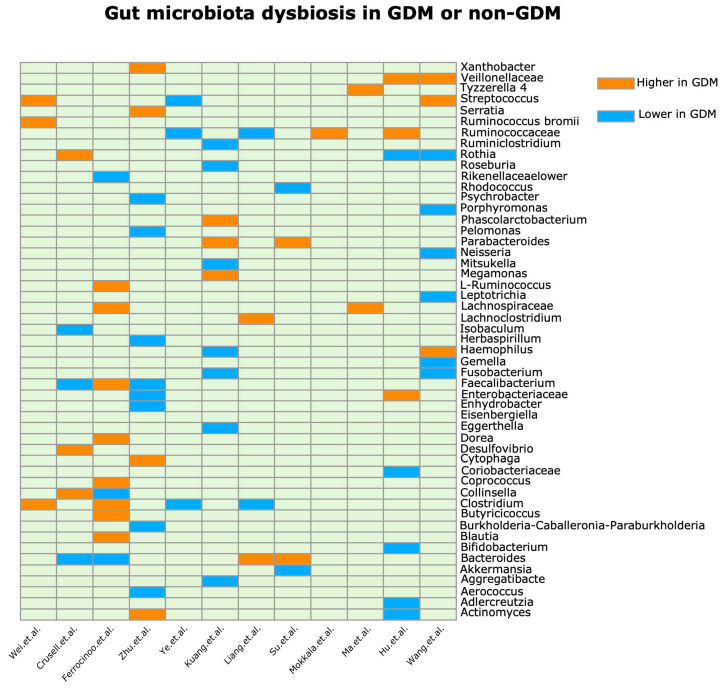
Changes in GDM and healthy pregnancy gut microbes (genera) [32,33,34,35,36,37,39,40,46,49,50,54].

In our review, we delineate marked alterations in the gut microbiota composition of pregnant women with GDM compared to healthy pregnant counterparts (Figure 2), exploring correlations and potential implications for GDM (Table 1, Figure 1). Notably, we noted significant shifts in the relative abundance of microbial genera including *Roseburia*, *Parabacteroides*, *Fusobacterium*, *Bacteroides*, and members of the *Lachnospiraceae* family. 

## 3. Microbial Metabolites in GDM

The gut microbes play pivotal roles in the catabolism of dietary fibers, proteins, and carbohydrates, with the capacity to ferment these macronutrients into short-chain fatty acids (SCFAs) such as propionate, butyrate, and acetate. SCFAs, generated from the fermentation of dietary fibers by gut microbes, provide energy to intestinal epithelial cells and shape immune responses [63]. Amino acid metabolites like indoles and phenyl derivatives influence neurotransmitter synthesis and brain function [64]. Bioactive amines including histamine and putrescine modulate immune reactions [65]. Lipid metabolites such as short-chain fatty acid amides enhance gut barrier function [59]. Secondary bile acid metabolites like deoxycholic acid suppress gut inflammation [60]. The production of vitamins especially vitamin K and B vitamins by gut microbes is also critical for nutritional absorption. SCFAs, along with other metabolic byproducts, furnish the host with essential energy and metabolites, which have significant modulatory effects on the host physiology and health [66,67]. Through the gut–brain axis, these microbial metabolites extensively impact the host immune, metabolic, and neurological systems and are implicated in various diseases. Further understanding the metabolic network of gut microbiota can lead to new therapeutic approaches targeting human diseases.

### 3.1. Role of Different SCFAs

SCFAs are the main products of dietary fiber fermentation by gut microbiota. Humans lack the enzymes needed to break down dietary fiber [68], which mostly passes through the upper digestive tract as the undigested forms and is fermented by anaerobic microbes in the cecum and colon [69]. *Faecalibacterium, Prevotella*, and some *Streptococcus* species are known producers of SCFAs in the gut [70]. A key mechanism of metabolic regulation by the gut microbiota is the production of SCFAs. Different gut microbes produce varying amounts of SCFAs, with the three main SCFAs produced in the gut being acetate, propionate, and butyrate [71]. 

Acetate is the most abundant SCFA in the human colon and is produced by Bifidobacterium and Lactobacillus fermenting fiber in the proximal colon. It is formed from acetyl-CoA via the Wood–Ljungdahl pathway [72]. Acetate acts on FFAR2 and FFAR3 in the human colon and increases fatty acid synthesis via epigenetic mechanisms (histone acetylation) [73]. Acetate is expressed at the mRNA level in pancreatic β-cell [72], which significantly inhibits insulin-induced glucose and fatty acid uptake in wild-type mouse adipocytes. FFAR2 (GPR43) inhibits insulin signaling via the G(I/O)βγ-PLC-PKC-PTEN pathway, reducing fat accumulation.

Butyrate is formed from two acetyl-CoA molecules, producing acetoacetyl-CoA, which is further converted to butyryl-CoA via β-hydroxybutyryl-CoA and crotonyl-CoA. A non-targeted metabolomics study by Sun et al. [62] on serum samples from GDM and NGT subjects found significant increases in 2-hydroxybutyric acid (2-HB) and l-α-amino butyric acid in GDM patients. Butyrate can activate AMPK in the liver, inhibiting gluconeogenic enzyme expression and promoting glycolysis. Furthermore, SCFAs can have an impact on blood glucose levels through their ability to regulate hormone secretion. They have been observed to promote insulin and GLP-1 secretion [74], the later inhibits glucagon secretion.

Propionate can be formed from Phosphoenolpyruvate (PEP) via the succinate pathway or the propionyl-CoA pathway, where lactate is reduced to propionate. Microbes can also produce propionate from hexoses (such as mannose and rhamnose) via the propanediol pathway [75]. A study by Sun et al. [62] found that consuming fiber-rich foods can alter the association between microbial characteristics and host glucose metabolism. Gut microbes digest dietary fiber into SCFAs, helping maintain normal glucose metabolism during pregnancy and preventing the onset of GDM.

SCFAs regulate the glycolysis and gluconeogenesis pathways and obstruct insulin signaling in peripheral tissues through the activation of GPCRs, resulting in hyperglycemia during pregnancy and diabetes [76]. SCFAs inhibit histone deacetylase activity and signal transduction via a group of free fatty acid receptors (GPR41, GPR43). After the discovery of SCFA receptors, GPR41 was renamed free fatty acid receptor 3 (FFAR3), and GPR43 was renamed FFAR2. FFAR2 and FFAR3 are found in human adipose tissue, colon, small intestine, and spleen [77]. Both FFAR2 and FFAR3 are associated with metabolic diseases and have become effective targets for the treatment of T2DM, asthma, cardiovascular diseases, and metabolic syndrome [78]. A study discovered that gut microbiota, SCFAs, and pancreatic β cells could potentially and collectively contribute to pregnancy glucose homeostasis mechanism in wild-type (WT) mice. During pregnancy, the measurable changes in circulating SCFA composition, increased FFAR2 expression, enhanced FFAR2 signaling, increased insulin secretion, and β cell proliferation compensate for pregnancy-related insulin resistance in together. A study by Wang et al. [79] investigated the composition of circulating SCFAs in pregnant women with GDM and showed its association with placental metabolism.

In summary, dietary fiber fermented by gut microbiota (e.g., *Bifidobacterium*, *Lactobacillus*) produces SCFAs including acetate, propionate, and butyrate, which act on FFAR2 and FFAR3 to inhibit histone deacetylases, suppress insulin signaling, activate AMPK, stimulate GLP-1 and insulin secretion, and enhance FFAR2 signaling in pregnancy to stimulate insulin release and pancreatic β-cell proliferation, thereby maintaining glucose homeostasis in gestational diabetes.

### 3.2. Role of Bile Acids

Elevated total bile acid levels in early pregnancy are positively associated with risk of GDM, while primary bile acids, cholic acid and chenodeoxycholic acid, are negatively correlated with GDM, and secondary bile acids [80], lithocholic acid and deoxycholic acid, are positively correlated with GDM risk [81]. Bile acids synthesized primarily in the liver can activate nuclear receptors FXR and TGR5 to regulate glucose and energy metabolism [82]. Gut microbiota can transform bile acids through reactions like dehydrogenation, altering enterohepatic circulation of bile acids [83] and generating secondary bile acid ligands for FXR and TGR5 [84], which has been shown experimentally to modulate host lipid and glucose metabolism [85]. The gut microbiota–bile acid axis is a key mechanism, whereby microbial bile salt hydrolases mediate bile acid metabolism and signaling [86], and alterations in bile acid profiles impact insulin sensitivity, lipid profiles, gut peptide release, and other aspects of host metabolism.

Accumulating evidence demonstrates significant alterations in the gut microbiota composition of GDM patients. For instance, *Bifidobacterium* [87] and *Lactobacillus* [88], which produce secondary bile acids, are decreased in the gut of GDM patients, whereas bile acid 7α-dehydroxylating bacteria including *Clostridium* [89], *Eubacterium* [90], and *Ruminococcin* are increased. These changes indicate imbalanced gut microbiota featured by altered populations of bacteria that promote or inhibit bile acid biotransformation in GDM patients, implying that dysregulation of the gut microbiota–bile acid axis may be a critical link in the pathogenesis of GDM.

### 3.3. Trimethylamine N-Oxide, TMAO

Trimethylamine N-oxide (TMAO) has been closely linked to various metabolism-related diseases. Research shows a positive correlation between TMAO levels and diabetes [91]. Dambrova et al.’s study reveals that TMAO levels are significantly higher in type 2 diabetes patients compared to non-diabetic controls [92]. Additionally, a randomized controlled trial confirmed that TMAO production is associated with gut microbiota composition [93]. Gut microbes play a key role in TMAO generation [94,95]. Dietary choline and phosphatidylcholine may undergo metabolism by the gut microbiota, resulting in the production of trimethylamine (TMA) [96,97], which is subsequently oxidized to TMAO in the liver. In terms of mechanisms, Chen et al. [98] and Seldin et al. [99] demonstrated that TMAO could activate MAPK, NF-κB and other signaling cascades and ROS production, consequently eliciting inflammatory responses. Studies by Dambrova et al. [92] and Randrianarisoa et al. [91] have demonstrated TMAO’s direct effect on pancreatic β cells, insulin release inhibition, and positive correlation with dysregulated blood glucose levels. In conclusion, TMAO may be involved in the pathogenesis of GDM by inducing inflammation and disrupting the insulin pathway. Thus, modulation of the gut microbiota is a potential way to reduce TMAO levels and disease risk.

Abnormalities in the metabolism of Palmitoylamides (PAs) and N-acetylglucosamine (GlcNAc) have been reported in patients with GDM. Lappas et al. [100] found that plasma concentrations of linoleoyl amide, arachidonoyl amide, and PAs were significantly reduced in the GDM group. Some PAs such as oleoylethanol amide can mimic GLP-1 and promote insulin release from pancreatic β cells [101]. PAs may induce insulin secretion by activating spiral ubiquitin-dependent receptors and GPR119 [102]. Additional research has verified that *Bifidobacterium*, an important genus of gut microbiota, is among the significant producers of GlcNAc [103]. They secrete extracellular agglomerates to extract aminoglucose from food and acetylate it to produce GlcNAc [104]. The GlcNAc that is produced can activate the downstream cAMP-PKA pathway by binding to the carbohydrate sensing receptor GPR92 on intestinal L cells [105], leading to the stimulation of GLP-1 synthesis and release. Additionally, by inducing short-chain fatty acid production in the intestinal mucosa through gut microbiota–epithelial cell interactions, GlcNAc can indirectly promote the secretion of GLP-1. In summary, the metabolic pathway through which *Bifidobacterium* and other gut microbiota stimulate GLP-1 release by producing GlcNAc has been elucidated. Changes in gut microbiota composition and function in GDM patients, such as reduced *Bifidobacterium* [33] and *Lactobacillus* [35], may cause modifications in the synthesis and transformation of metabolites, such as PAs and GlcNAc. These two compounds have GLP-1-like effects and can enhance insulin sensitivity.

**Figure 3:**
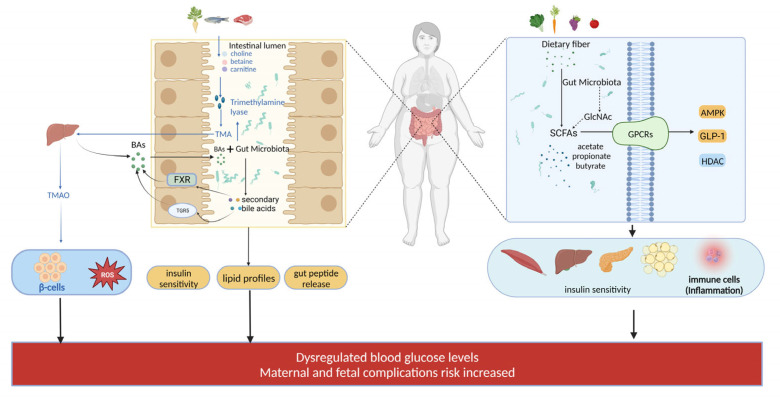
Gut microbes and their metabolites: possible mechanisms of GDM. TMAO Production: Gut microbiota significantly affect TMAO production, as they convert dietary choline and phosphatidylcholine into TMA, which the liver subsequently transforms into TMAO. TMAO activates MAPK and NF-κB pathways, leading to ROS production and inflammation. TMAO also inhibits insulin release, contributing to glucose dysregulation. Bile Acid Modification: Gut microbiota modify bile acids via enzymatic reactions, altering their profiles and impacting lipid and glucose metabolism. The gut microbiota–bile acid axis plays a crucial role, affecting insulin sensitivity, lipid profiles, and gut peptide release. SCFAs: Anaerobic microbes ferment undigested dietary fiber, producing SCFAs (acetate, propionate, and butyrate). SCFAs interact with FFAR2 and FFAR3 receptors, influencing glycolysis, gluconeogenesis, and insulin signaling. They help maintain glucose homeostasis during pregnancy, mitigating insulin resistance. GlcNAc and Metabolic Pathways: Specific pathways activate GLP-1 and promote its secretion indirectly through SCFA production in the intestinal mucosa.

In addition to SCFAs, bile acids, trimethylamine, and N-acetyl amino sugars, some studies have also identified other related metabolic products [106]. Amino acid metabolic products, in particular, tyrosine and tryptophan metabolites have been shown to have an impact on GLP-1 synthesis [107]. The decreased levels of indole compounds, metabolites derived from tryptophan through microbial decarboxylation and dehydrogenation reactions, in maternal serum during early and mid-gestation are associated with an increased risk of GDM [108], which may be attributed to reduced populations of tryptophan-metabolizing bacteria like *Bifidobacterium* [87] and *Lactobacillus* [88] in the gut microbiota of GDM patients, consequently leading to impaired synthesis of indole-3-propionic acid and secretion of incretins like GLP-1 and GLP-2, further aggravating insulin resistance in GDM [109]. Furthermore, γ-aminobutyric acid can affect insulin sensitivity through the GPR41/43 pathway [65]. Meanwhile, protein fermentation products, such as fecal p-cresol, have been found to improve insulin sensitivity, and fecal indole has been shown to inhibit inflammatory response [110]. Bioactive amines such as histamine and acetylcholine may have an impact on glucose homeostasis. Additionally, intestinal microbiota-produced phenyl propionic acid can chelate iron, potentially affecting glucose absorption [108]. In summary, various intestinal microbial metabolites participate in the pathogenesis of diabetes by regulating insulin secretion and sensitivity (Figure 3). Therefore, the gut microbiota’s integral role and its concomitant metabolic signatures underscore some pathophysiological landscape of GDM.

## 4. Microbes and Microbial Metabolites in the Prevention and Treatment of GDM

Synthetically, studies have suggested that the gut microbiota altered composition and metabolic output may be instrumental in the metabolic perturbations of glucose regulation seen in GDM, which underscores the potential of microbe-centric interventions in mitigating GDM. Probiotics harboring *Lactobacillus* and *Bifidobacterium* strains demonstrate efficacy in enhancing insulin sensitivity and glucose metabolism, with supplemental administration improving clinical biomarkers in GDM [111,112]. Selected strains within these probiotics not only attenuate systemic inflammation but also modulate the gut and vaginal microbiomes, contributing to a reduced GDM risk and less pronounced maternal weight gain [113].

Dietary prebiotics, serve as fermentable substrates that foster beneficial microbial populations, thereby promoting glucose homeostasis in GDM contexts [114]. Moreover, microbial metabolites, particularly SCFAs like butyrate and propionate, are implicated in enhancing insulin sensitivity and suppressing hepatic gluconeogenesis, alongside their noted anti-inflammatory properties, all of which are pivotal in GDM management [115]. The combined use of multi-strain probiotics, prebiotics, and symbiotic appears to hold additive or synergistic potential in regulating glucose metabolism and aiding in weight management during GDM [116].

### 4.1. Probiotic Supplements

Recent randomized controlled trials have examined the efficacy of probiotic supplementation for improving outcomes in pregnant women with GDM [117,118]. Some studies have found certain probiotic strains to modestly improve select biomarkers, including decreases in fasting plasma glucose, inflammatory markers like high-sensitivity C-reactive protein (hs-CRP), and lipid parameters such as triglycerides and LDL cholesterol [116]. However, the majority of RCTs have not demonstrated significant improvements in clinical maternal or neonatal outcomes, including the need for pharmacological therapy, cesarean section rates, gestational weight gain, preeclampsia risk, and birth weight or gestational age of the newborn [119].

The effects appear to be strain-specific, with trials using various strains and combinations of *Bifidobacterium*, *Lactobacillus*, and other genera with mixed results. Limitations of these RCTs include small sample sizes (*n* < 100), short intervention durations (6–12 weeks), and lack of standardized probiotic dosing [120,121]. Overall, current evidence does not strongly support probiotics as a standalone therapy for GDM management, but they may provide subtle metabolic benefits that warrant further mechanistic study. Larger, longer-term RCTs with optimized probiotic formulations are needed to determine if probiotics can significantly improve maternal glycemic control and neonatal health outcomes in GDM. Current guidelines do not recommend probiotics for GDM, but future research may clarify their clinical utility as adjuvant or preventative therapy.

### 4.2. Prebiotic Supplementation

Prebiotics, indigestible food components, foster the proliferation of beneficial gut microbiota by serving as substrates for select bacteria [122]. These substrates, including inulin from chicory root and fructo-oligosaccharides (FOS) found in bananas and onions, resist gastric digestion and are fermented in the colon, selectively enhancing populations of health-promoting bacteria such as *Bifidobacterial* [123] and *Lactobacilli*. Such activities not only support the gut ecosystem but also confer systemic health benefits, including improved digestive health and immunity [124].

In the context of GDM, prebiotic supplementation is gaining traction as a potential therapeutic avenue. Non-digestible fibers like inulin, FOS, and galactooligosaccharides (GOS) have been shown to modulate gut microbiota favorably [125], which can lead to improved glucose metabolism and insulin sensitivity [126]. Mechanistically, prebiotics enhance SCFAs production, which, alongside shifts in microbial composition, contributes to anti-inflammatory effects and improved intestinal barrier integrity [127]. Clinical evidence points to increased levels of beneficial *Lactobacilli* and elevated SCFAs biomarkers in response to prebiotic intake in GDM patients [118]. SCFAs, particularly butyrate, serve not only as an energy source for enterocytes but also as modulators of inflammation and insulin response [128]. Further, prebiotics elevate other SCFAs like acetate and propionate, and metabolites such as indoles and polyamines, which have been implicated in improved metabolic outcomes [129].

BAs, traditionally recognized for their role in lipid digestion and vitamin absorption, have gained prominence as key metabolic signaling molecules. Through activation of the farnesoid X receptor (FXR) and the G protein-coupled bile acid receptor (TGR5), BAs exert regulatory control over diverse metabolic processes, with dysregulated BAs signaling emerging as a factor in GDM. Prebiotic interventions are posited to reshape gut microbiota composition, leading to several BA-related metabolic improvements: By fostering the growth of BA-metabolizing bacteria, prebiotics may alter the BA pool, transitioning primary BAs to secondary forms, with implications for pool composition and size [130]. Secondary BAs, modulated by prebiotic intake, can differentially engage BA receptors, including FXR and TGR5—receptors integral to glucose and lipid homeostasis [131]. Enhancing BA excretion fosters hepatic synthesis from cholesterol, potentially offering a therapeutic benefit in GDM-associated dyslipidemia [132]. Prebiotic-induced microbial shifts may bolster intestinal barrier function and temper inflammation, contributing to improved insulin sensitivity [133]. Yet, pregnancy presents a unique physiological milieu, with intrinsic alterations in BA metabolism necessitating cautious therapeutic interventions [134]. While prebiotic supplementation offers a novel modality for BA modulation in GDM, its integration into clinical practice mandates a foundation of solid empirical evidence and nuanced clinical judgment.

TMAO, a byproduct of dietary choline, lecithin, and L-carnitine metabolized by gut microbiota into TMA, and subsequently oxidized in the liver, has been implicated in heightened cardiovascular disease (CVD) risk due to its proatherogenic properties [135,136]. In the milieu of GDM—a condition inherently associated with increased cardiovascular morbidity—the augmented levels of TMAO may contribute to an intensified risk profile [89]. Interventions via prebiotic supplementation to modulate TMAO concentrations present an attractive therapeutic strategy, albeit one that requires careful consideration due to the altered physiological landscape of pregnancy.

The influence of prebiotics on TMAO levels within the GDM framework could theoretically extend through multiple avenues: the alteration of intestinal microbiota composition to curtail the prevalence of TMA-producing microbes [137]. The attenuation of choline, lecithin, and L-carnitine metabolism into TMA by selective bacterial communities. The amelioration of metabolic parameters, including glycemic regulation and lipid profile optimization, which could indirectly influence TMAO pathways [47]. Notwithstanding these prospective benefits, the dynamics among prebiotic interventions, gut microbial ecology, TMAO synthesis, and metabolic health during pregnancy are not thoroughly understood. Research delineating the adverse impact of TMAO has been predominantly conducted in non-pregnant cohorts [138], and gestational adjustments in renal physiology could modulate TMAO clearance, further complicating the extrapolation to a GDM context.

In light of these considerations, while prebiotic-driven TMAO modulation offers a theoretically viable route to mitigate cardiovascular complications in GDM, a rigorous scientific inquiry is essential. This should be aimed at elucidating the interconnections and establishing a safe and effective intervention protocol within the specialized context of maternal and fetal health. The revised paragraph emphasizes the potential of prebiotics in GDM management while calling for careful, pregnancy-specific research to understand and utilize the microbiome–TMAO relationship.

Despite these promising mechanisms, clinical data on the efficacy of prebiotics in GDM management exhibit some variability [139]. Therefore, while prebiotic supplementation shows potential in correcting dysbiosis and augmenting SCFA levels—thereby ameliorating glucose dysregulation and reducing GDM complications—the determination of the most effective prebiotic types and doses necessitates further investigation. Future studies must validate both the efficacy and safety of prebiotic interventions in GDM to establish definitive recommendations. In summary, targeted modulation of gut microbiota using probiotics, prebiotics, and microbiota-directed foods holds promise as an adjuvant GDM therapy, warranting further validation in larger, high-quality randomized controlled trials.

## 5. Conclusions and Future Perspectives

Our review reveals the links between gut dysbiosis, abnormal glucose metabolism, inflammation, and insulin resistance in GDM. Interestingly, it notes that some of the altered bacteria in the gut show similar trends in both the mother and her offspring, suggesting that the mother’s microbiome might be transmitted to the child. This transmission reflects how the gut microbiota of a GDM mother could influence the colonization process of her child’s gut microbiota. These insights not only deepen our understanding of the complex mechanisms underlying GDM but also open potential avenues for therapeutic interventions targeting the gut microbiome. While lifestyle interventions alone may not be sufficient for the management of GDM, targeted probiotic supplementation is a promising adjuvant therapy, although optimal formulations remain to be determined [117,118]. Current understanding of the complex gut microbiota–host interactions in GDM is limited by the small, ethnically homogeneous cohorts and conflicting results. Large, multi-ethnic studies using standardized microbiome workflows and integrated multi-omics approaches (e.g., meta transcriptomics, proteomics, metabolomics) through advanced bioinformatics are warranted to elucidate mechanisms linking specific microbes and functions to GDM pathogenesis. Such efforts can inform evidence-based diagnostic, preventive, and therapeutic strategies aimed at restoring gut microbial homeostasis to improve pregnancy outcomes in GDM.

## Figures and Tables

**Table 1:** Research on gut microbiota and its metabolic impact in GDM studies.

Title	Study Type	Timing of Analysis	Country and/or Ethnicity	Sample Size		Main Results		
				GDM	non = GDM	Microbial Abundance and Correlations	Inferred Functional	Gut Microbiota Profile in GDM Patients in Comparison to Controls (Phylum/Order/Family/Genus)
Mokkala et al. [34]	Prospective cohort study	12.9 weeks of gestation	Finland	15	60	*Ruminococcaceae* abundance is positively correlated with fasting glucose.	*Ruminococcaceae* may impede glucose homeostasis by affecting inflammation, insulin signaling, SCFA production, and energy harvest.	Genus: Increased abundance of an unidentified genus of Ruminococcaceae family in GDM patients.
Ma et al. [33]	Nested case–control study	10–15 weeks of gestation	Chinese Han ethnicity	98	98	*Eisenbergiella* and *Tyzzerella* are positively correlated with fasting glucose. *Parabacteroides, Parasutterella,* and *Ruminococcaceae* were negatively correlated with fasting glucose.	Predicted enrichment of sphingolipid metabolism, starch/sucrose metabolism pathways in GDM. Reduction in lysine biosynthesis and nitrogen metabolism pathways in GDM.	Genus: Higher *Eisenbergiella, Tyzzerella 4, Lachnospiraceae NK4A136* in GDM group. Higher *Parabacteroides, Megasphaera, Ruminococcaceae, Eubacterium* in controls.
Gomez et al. [31]	Cross-sectional study	mean 16 weeks of gestation	Australia, mostly Caucasian	18	70 (29 overweight, 41 obese)—18 developed GDM later	The genus *Collinsella* (phylum *Actinobacteria*) was positively correlated with insulin, C-peptide, HOMA-IR, triglycerides, and VLDL cholesterol. The genus *Coprococcus* (family *Lachnospiraceae*) was positively correlated with GIP. *Bacteroidaceae* positively and *Prevotellaceae* negatively correlated with ghrelin.	Maternal metabolic hormones.	Increased *Actinobacteria*.
Su et al. [40]	Cross-sectional study	24–28 weeks of gestation	Shanghai, China; Han ethnicity	21	32	*Bacteroidetes* positively correlated with 1hPG, FINS, 1hPIN, and HOMA-IR. *Proteobacteria, Actinobacteria,* and *Verrucomicrobia negatively* correlated with 1hPG. *Ruminococcaceae UCG014* negatively correlated with glycemic traits. *Incertae Sedis* positively correlated with FPG and 1hPG. *Akkermansia* negatively correlated with 1hPG.	GDM patients had higher microbial gene functions related to amino sugar and nucleotide sugar metabolism. *Bacteroides* genus positively correlated with amino sugar/nucleotide sugar metabolism. Controls had higher gene functions related to two-component system, ABC transporters, and transporters	Phyla: *Bacteroidetes* higher, and *Proteobacteria*, *Actinobacteria*, *Verrucomicrobia* lower in GDM. Genera: *Bacteroides Parabacteroides* higher, *Akkermansia Rhodococcus* lower in GDM.
Wang et al. [54]	Case–control study	24–28 weeks of gestation	China	59	48	*Lachnospiraceae* OTUs positively correlated with glucose levels. *Enterobacteriaceae* OTUs negatively correlated with glucose levels.	Disturbances in fecal and urinary metabolites related to amino acid and carbohydrate metabolism.	At the family level, there was increased *Lachnospiraceae* and decreased *Enterobacteriaceae* and *Ruminococcaceae* in GDM.
Wei et al. [37]	Case–control study	24–28 weeks of gestation	China	15	18	*S. infantis* positively correlated with glucose levels.	-	GDM patients had increased *Ruminococcus bromii, Clostridium colinum,* and *Streptococcus infantis* at genus level compared to controls.
Chen et al. [49]	Cross-sectional study	24–28 weeks of gestation	China	30	28	*Prevotella* and *Romboutsia* genera negatively correlated with 2 h glucose in GDM *Aureimonas, Kosakonia* species positively correlated and *Peptostreptococcus* negatively correlated with fasting glucose in GDM.	Depletion of beneficial acetate and lactate-producing bacteria like *Bifidobacterium*, and butyrate-producing bacteria like *Eubacterium* suggests beneficial SCFA production may be reduced in GDM.	At the genus level, 54 differentially abundant taxa identified, with 42 genera depleted in GDM (e.g., *Prevotella, Romboutsia*). GDM had lower levels of beneficial bacteria like *Bifidobacterium, Eubacterium,* and *Prevotella* species. GDM was enriched in *Blautia hydrogenotrophica* and some *Corynebacterium, Lactobacillus* species.
Liang et al. [36]	Case–control study	Second trimester	China	35	25	*Ruminococcaceae_UCG-002, Ruminococcaceae_UCG-005, Clostridium_sensu_stricto_1,* and *Streptococcus* were more abundant in controls compared to GDM patients. *Bacteroides* and *Lachnoclostridium* were more abundant in GDM patients compared to controls. *Paraprevotella, Roseburia, Faecalibacterium*, and *Ruminococcaceae_UCG-002* were negatively correlated with glucose. *Ruminococcaceae_UCG-002* was negatively correlated with HbA1c. *Bacteroides* was positively correlated with glucose. *Sutterella, Oscillibacter,* and *Bifidobacterium* were positively correlated with GLP-1 levels.	*Paraprevotella, Roseburia,* and *Faecalibacterium* negatively correlated with glucose. *Bacteroides* positively correlated with glucose. *Sutterella, Oscillibacter, Bifidobacterium* was positively associated with GLP-1.	At the phylum level, *Firmicutes, Bacteroidetes, Proteobacteria,* and *Actinobacteria* were the main phyla in both groups. *Firmicutes* and *Bacteroidetes* decreased and increased in GDM, respectively. At the family level, *Ruminococcaceae, Lachnospiraceae,* and *Christensenellaceae* were more abundant in NGT. *Bacteroidaceae* increased in GDM. At the genus level, *Ruminococcaceae_UCG-002, Ruminococcaceae_UCG-005,* and *Clostridium_sensu_stricto_1* were lower in GDM, while *Bacteroides* and *Lachnoclostridium* were higher in GDM.
Sun et al. [62]	Nested case–control study	24–28 weeks of gestation	China	120	120	Fecal short-chain fatty acids like propionate and butyrate increased more from first to second trimester in controls versus GDM patient.	Microbial pathways related to dietary fiber fermentation (e.g., mannan degradation) were lower in abundance in GDM patients.	At the phylum level, the *Firmicutes*-to-*Bacteroidetes* (F/B) ratio decreased with advancing gestation in controls but not in GDM patients. Depleted in GDM: *Ruminococcus bromii, Alistipes putredinis, Bacteroides ovatus, Bifidobacterium dentium.* Enriched in GDM: *Escherichia coli, Fusobacterium mortiferum, Bacteroides massiliensis, Eubacterium ramulus, Anaerostipes hadrus*.
Ferrocinoo et al. [46]	Prospective cohort study	24–28 weeks of gestation, 38 weeks of gestation	Italy	41	0	Predicted metagenomic analysis suggested enrichment of pathways involved in carbohydrate metabolism and LPS biosynthesis in third trimester.	*Faecalibacterium* was significantly associated with fasting glucose; *Collinsella* (directly) and *Blautia* (inversely) were associated with insulin and with homeostasis model assessment of insulin resistance, while *Sutterella* was associated with C-reactive protein levels. Consistent with this latter association, the predicted metagenomes showed a correlation between those taxa and inferred KEGG genes associated with lipopolysaccharide biosynthesis	At the phylum level, Firmicutes increased and Bacteroidetes and Actinobacteria decreased from 2nd to third trimester. At the genus level, *Blautia, Butyricicoccus, Clostridium, Coprococcus, Dorea, Faecalibacterium, L-Ruminococcus*, and *Lachnospiraceae* increased, while *Bacteroides, Collinsella* and *Rikenellaceae* decreased.
Kuang et al. [35]	Cross-sectional study	21–29 weeks of gestation	china	43	81	Gut microbiota abundance correlated with glucose levels: Positively correlated genera: Parabacteroides, Megamonas, Klebsiella, etc. Negatively correlated genera: Alistipes, Bifidobacterium, Eubacterium, etc.	Inferred functional characters in GDM: Increased membrane transport, energy metabolism pathways, LPS and PTS systems; Decreased amino acid metabolism pathways.	Order: Decreased *Clostridiales* in GDM Family: Decreased *Coriobacteriaceae* in GDM Genus: Increased *Parabacteroides, Megamonas,* and *Phascolarctobacterium* in GDM; Decreased *Ruminiclostridium, Roseburia, Eggerthella, Fusobacterium, Haemophilus, Mitsukella,* and *Aggregatibacter* in GDM
Crusell et al. [47]	Cross-sectional study	Analysis in third trimester and 8 months postpartum	Danish white pregnant women	In third trimester 50, postpartum 43	In third trimester157, postpartum79	*Collinsella* positively correlated with fasting glucose (adjusted for BMI) *Butyricicoccus* negatively correlated with insulin sensitivity (adjusted for BMI). *Prevotella* and *Faecalitalea* positively correlated with 2 h glucose (adjusted for BMI).	Two species of *Blautia* were associated with lower levels of plasma hsCRP, pointing to the occurrence of various subspecies of *Blautia* with opposite functionality related to host metabolism.	Phylum *Actinobacteria* higher in GDM. Genera *Collinsella, Rothia, Desulfovibrio* higher in GDM. Genera *Faecalibacterium*, *Bacteroides, Isobaculum* lower in GDM. 17 species-level OTUs differentially abundant between GDM and controls.

HOMA-IR: homeostatic model assessment of insulin resistance; VLDL: very low density lipoprotein; GIP: gastric inhibitory polypeptide; 1hPG: 1 h post-glucose; FINS: fasting insulin; 1hPIN: 1 h post-ingestion; FPG: fasting plasma glucose; OTUs: operational taxonomic units; GDM: gestational diabetes mellitus; HbA1c: hemoglobin A1c; GLP-1: glucagon-like peptide-1; LPS: lipopolysaccharides; BMI: body mass index; SCFA: short-chain fatty acids; ABC transporters: ATP-binding cassette transporters; KEGG: Kyoto Encyclopedia of Genes and Genomes; PTS: phosphotransferase system; hsCRP: high-sensitivity C-reactive protein.
